# Paclitaxel-Coated Zilver PTX Drug-Eluting Stent Treatment Does Not Result in Increased Long-Term All-Cause Mortality Compared to Uncoated Devices

**DOI:** 10.1007/s00270-019-02324-4

**Published:** 2019-09-09

**Authors:** Michael D. Dake, Gary M. Ansel, Marc Bosiers, Andrew Holden, Osamu Iida, Michael R. Jaff, Aaron E. Lottes, Erin E. O’Leary, Alan T. Saunders, Marc Schermerhorn, Hiroyoshi Yokoi, Thomas Zeller

**Affiliations:** 1 The University of Arizona, Roy P. Drachman Hall Building, B207, 1295 North Martin Avenue, P.O. Box 210202, Tucson, AZ 85721-0202 USA; 2 Department of Medicine, Ohio Health/Riverside Methodist Hospital, Columbus, OH USA; 3 Foundation of Cardiovascular Research and Education, Münster, Germany; 4 Department of Interventional Radiology, Auckland City Hospital, Auckland, New Zealand; 5 Cardiovascular Center, Kansai Rosai Hospital, Amagasaki, Japan; 6 Harvard Medical School, Boston, MA USA; 7 Cook Research Incorporated, West Lafayette, IN USA; 8 Division of Vascular Surgery, Department of Surgery, Beth Israel Deaconess Medical Center, Boston, MA USA; 9 Department of Cardiovascular Medicine, Fukuoka Sanno Hospital, Fukuoka, Japan; 10 Universitaets-Herz-Zentrum Freiburg – Bad Krozingen, Bad Krozingen, Germany

**Keywords:** Paclitaxel, Drug-eluting stent, Bare metal stent, Percutaneous transluminal angioplasty, Mortality, Peripheral artery disease

## Abstract

**Purpose:**

Patient-level data from two large studies of the Zilver PTX drug-eluting stent (DES) with long-term follow-up and concurrent non-drug comparator groups were analyzed to determine whether there was an increased mortality risk due to paclitaxel.

**Methods:**

Data from the Zilver PTX randomized controlled trial (RCT) and Zilver PTX and bare metal stent (BMS) Japan post-market surveillance studies were analyzed. Five-year follow-up is complete in both DES studies; follow-up for the BMS study was limited to 3 years and is complete. Kaplan–Meier analyses assessed mortality. A Cox proportional hazards model identified significant factors related to mortality.

**Results:**

In the RCT, there were 336 patients treated with the DES and 143 patients treated with percutaneous transluminal angioplasty (PTA) or BMS. In Japan, there were 904 DES patients and 190 BMS patients. There was no difference in all-cause mortality for the DES compared to PTA/BMS in the RCT (19.1% DES versus 17.1% PTA/BMS through 5 years, *p *= 0.60) or Japan (15.8% DES versus 15.3% BMS through 3 years, *p *= 0.89). Cox proportional hazard models revealed that age, tissue loss, and congestive heart failure were significantly associated with mortality in the RCT, and critical limb ischemia, age, renal failure, and gender were significantly associated with mortality in Japan (all *p *< 0.05). Neither treatment with Zilver PTX (*p *= 0.46 RCT, *p *= 0.49 Japan) nor paclitaxel dose (*p *= 0.86 RCT, *p *= 0.07 Japan) was associated with mortality.

**Conclusion:**

Analyses of the Zilver PTX patient-level data demonstrated no increase in long-term all-cause mortality.

**Level of Evidence:**

Zilver PTX RCT: Level 1, randomized controlled trial; Japan PMS studies: Level 3, post-market surveillance study.

**Electronic supplementary material:**

The online version of this article (10.1007/s00270-019-02324-4) contains supplementary material, which is available to authorized users.

## Introduction

An endovascular-first approach is commonly used to treat symptomatic femoropopliteal peripheral artery disease (PAD) [1, 2]. Over nearly a decade, drug–device combination technologies using the antiproliferative agent, paclitaxel, have been developed to reduce restenosis rates compared to percutaneous transluminal angioplasty (PTA) or bare metal stents (BMS) [3–11].

Recently, a meta-analysis that grouped both drug-eluting stents (DES) and drug-coated balloons (DCB) together indicated a higher incidence of late all-cause mortality for paclitaxel-based devices compared to uncoated PTA or BMS at 2 years up to 5 years [12]. This difference in mortality was attributed directly to paclitaxel although no etiology was postulated for the increased rate. The authors reported a dose-dependent relationship in the mortality rate that was based on the dose density of each device rather than the actual amount of paclitaxel coated on the device.

The authors of the meta-analysis used data available in publications or other public online resources. For the Zilver PTX DES, the published data were an intent-to-treat summary analysis, which accounted for patients’ treatment at the time of enrollment. However, due to the unique trial design of the Zilver PTX randomized controlled trial (RCT), patients were not limited to stent placement only at enrollment. To evaluate the long-term mortality rate of the DES, the patient-level data from the Zilver PTX RCT were analyzed based on treatment actually received, and the results for patients treated with the DES were compared to patients treated only with PTA and/or BMS. Supporting evidence from the Japan Zilver PTX and Zilver BMS post-market studies (PMS) provides long-term mortality information from a broader patient population.

## Methods

### Randomized Controlled Trial

The Zilver PTX RCT was a prospective, multinational, randomized study comparing the safety and effectiveness of the polymer-free, paclitaxel-coated Zilver PTX DES (Cook Medical, Bloomington, IN, USA) to PTA and provisional BMS placement in patients with femoropopliteal PAD. Study characteristics have been previously described [3] and are summarized in Online Resource 1. Five-year follow-up is complete, and results have been published [3–5]. Patients with symptomatic PAD involving the above-the-knee femoropopliteal arteries were initially randomized to PTA or to stent placement with the paclitaxel-coated Zilver PTX DES. Patients with acute PTA failure (e.g., ≥ 30% residual stenosis remaining after minimum of additional 2- to 3-min balloon inflation) underwent a secondary randomization and were treated with either the DES or a BMS (Zilver, Cook Medical, Bloomington, IN, USA). PTA patients and BMS patients who required re-intervention within the first year post-procedure were permitted to cross over to treatment with the DES. Thus, there were three opportunities to receive a DES: two via protocol-specified randomization and the third via crossover due to subsequent failure of an initially successful treatment (note, patients had reached the study endpoint for efficacy). Recognizing missing data as a limitation, vital status for patients who did not originally complete the study was ascertained; all available vital status data are included in the current mortality analyses.

### Japan Post-market Surveillance Study

Post-market data were collected from a prospective, multicenter registry of the Zilver PTX DES as well as concurrent prospectively enrolled patients treated with the Zilver BMS (Online Resource 1).

The Japan DES PMS had no exclusion criteria and enrolled consecutive patients with symptomatic PAD involving the above-the-knee femoropopliteal arteries. Safety and effectiveness of the DES were evaluated in real-world patients with complex femoropopliteal artery lesions through 5 years [13, 14]. Follow-up in the study is complete.

The concurrent Japan BMS PMS also had no exclusion criteria and enrolled consecutive patients with symptomatic PAD involving the above-the-knee femoropopliteal arteries. Patients who were enrolled in the BMS study but who also had a DES placed (*n* = 18) were excluded from the current analysis. Follow-up in the BMS study was only required through 3 years and is complete.

### All-Cause Mortality and Adverse Events

All deaths reported during active study follow-up were adjudicated by an independent clinical events committee to determine the cause of death and relatedness to the procedure or device.

### Statistical Analysis

Analyses were performed using SAS 9.4 (SAS Institute, Cary, NC, USA). All comparisons of the DES group to the PTA/BMS group were made based on treatment actually received. Continuous variables were summarized with means and standard deviations, with *p* values calculated using the standard *t* test. Categorical variables were reported as counts and percentages, with *p* values calculated using Fisher’s exact test. Kaplan–Meier analyses were performed to assess mortality. In the RCT analyses, day 0 was set as the day of crossover to DES treatment. *p* values for the Kaplan–Meier analyses were calculated using the log-rank test. Cox proportional hazard models for paclitaxel treatment (binary) and paclitaxel dose (continuous; amount of paclitaxel received by each patient) were performed using the relevant covariates expected to have a potential impact on mortality. The model incorporated all covariates simultaneously to ascertain whether there was a potential confounding factor that could influence mortality beyond the factor in question, i.e., DES versus PTA/BMS. The patient-level data used for the RCT analyses presented here is available on the following website: https://www.cookmedical.com/peripheral-intervention/paclitaxel/.

## Results

### Randomized Controlled Trial

**Fig. 1:**
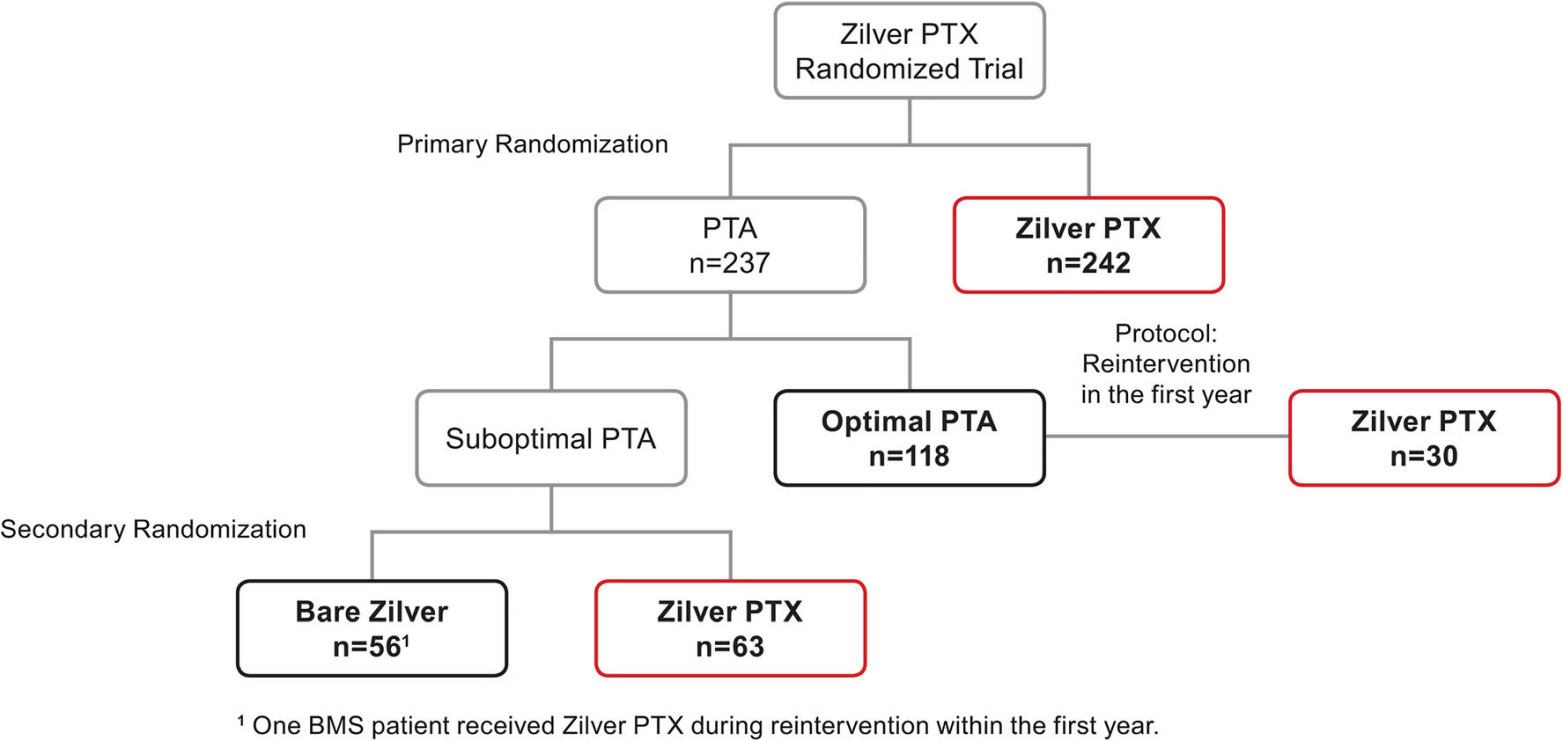
Patient flow diagram for patients enrolled in the Zilver PTX RCT. Patient flow diagram based on actual treatment, with DES patients shown in red and PTA and BMS patients shown in black. Protocol specified that patients who experienced re-intervention within the first year could cross over to DES treatment. RCT, randomized controlled trial; DES, drug-eluting stent; PTA, percutaneous transluminal angioplasty; BMS, bare metal stent

**Fig. 2:**
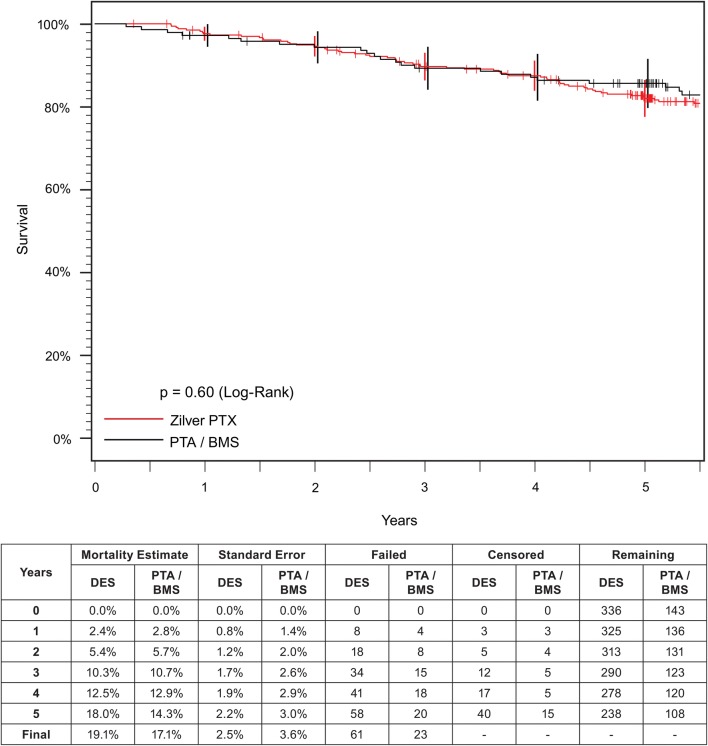
Kaplan–Meier survival analysis for patients in the Zilver PTX RCT. Long-term mortality analysis shows no difference between the DES (red curve) and PTA/BMS (black curve). RCT, randomized controlled trial; DES, drug-eluting stent; PTA, percutaneous transluminal angioplasty; BMS, bare metal stent

Of the 479 patients enrolled in the RCT, 237 patients were included in the primary PTA group and 242 patients were included in the primary DES group (Fig. 1). However, 63 patients who were randomized to primary PTA treatment received the DES following acute PTA failure, and an additional 31 patients received the DES as treatment for failure of their original therapy within the first year. As a result, 94 patients who were initially randomized to the primary PTA group were actually treated with the DES (40% of the primary PTA group received a DES). In total, 336 patients were treated with a DES and 143 patients were treated only with PTA and/or BMS (Fig. 1). Baseline patient and lesion characteristics are summarized in Table 1. Five-year mortality data were available for 94% of patients. There were 61 deaths in the DES group and 23 deaths in the PTA/BMS group through 5 years; none were adjudicated as procedure- or device-related. There was no difference in the 5-year Kaplan–Meier estimates for all-cause mortality for the DES group compared to the PTA/BMS group (19.1% vs. 17.1%, log-rank *p *= 0.60; Fig. 2). There were no significant differences between the DES and PTA/BMS groups in causes of death (Table 2) or adverse events (Online Resource 2).

**Table 1:** Zilver PTX RCT demographics and baseline lesion characteristics

Characteristic	DES	PTA/BMS	*p* value
Age (years)	67.6 ± 9.6 (336)	68.2 ± 11.2 (143)	0.58
Gender			0.60
Male	65.8% (221/336)	62.9% (90/143)	
Female	34.2% (115/336)	37.1% (53/143)	
Ethnicity			0.96
Asian	12.8% (38/298)	14.0% (17/121)	
Black/African-American	12.1% (36/298)	10.7% (13/121)	
Hispanic/Latino	6.7% (20/298)	5.8% (7/121)	
White/Caucasian	68.5% (204/298)	69.4% (84/121)	
Body mass index	28.3 ± 5.2 (336)	28.4 ± 6.0 (143)	0.87
Diabetes	45.2% (152/336)	46.9% (67/143)	0.76
Diabetes type			0.54
Type I	15.8% (24/152)	11.9% (8/67)	
Type II	84.2% (128/152)	88.1% (59/67)	
Hypercholesterolemia	75.0% (252/336)	67.8% (97/143)	0.12
Hypertension	87.5% (294/336)	80.4% (115/143)	0.049
Carotid artery disease	19.0% (64/336)	18.9% (27/143)	> 0.99
Renal disease	9.5% (32/336)	11.9% (17/143)	0.51
Pulmonary disease	18.5% (62/336)	14.7% (21/143)	0.36
Congestive heart failure	11.3% (38/336)	11.2% (16/143)	> 0.99
Previous cardiac arrhythmia	9.8% (33/336)	16.1% (23/143)	0.06
Previous MI	20.5% (69/336)	15.4% (22/143)	0.21
Smoking status			0.54
Never	14.6% (49/336)	15.4% (22/143)	
Quit	54.2% (182/336)	51.7% (74/143)	
Still smokes	31.3% (105/336)	32.2% (46/143)	
Unknown	0% (0/336)	0.7% (1/143)	
Rutherford 0–3	92.0% (309/336)	89.4% (126/141)	0.38
Rutherford 4–6	8.0% (27/336)	10.6% (15/141)	
Lesion length (mm)	55.8 ± 41.1 (354)	51.8 ± 39.8 (149)	0.32
Total occlusion	31.1% (110/354)	28.2% (42/149)	0.60
Proximal RVD (mm)	5.1 ± 1.0 (350)	5.0 ± 0.9 (148)	0.65
Distal RVD (mm)	5.0 ± 1.0 (350)	5.0 ± 1.1 (148)	0.98
MLD in lesion (mm)	1.0 ± 0.9 (350)	1.1 ± 0.9 (148)	0.48
Percent diameter stenosis (%)	79.5 ± 17.1 (350)	78.5 ± 16.7 (148)	0.59
Calcification			0.08
None	2.8% (10/354)	5.4% (8/149)	
Little	30.2% (107/354)	38.3% (57/149)	
Moderate	30.5% (108/354)	22.1% (33/149)	
Severe	36.4% (129/354)	34.2% (51/149)	
Other stenosis in artery			0.049
None	51.1% (180/352)	50.3% (75/149)	
≤ 50%	32.1% (113/352)	40.3% (60/149)	
> 50%	16.8% (59/352)	9.4% (14/149)	
Inflow tract stenosis			0.023
None	41.5% (146/352)	47.0% (70/149)	
≤ 50%	39.5% (139/352)	43.6% (65/149)	
> 50%	19.0% (67/352)	9.4% (14/149)	

Numbers in parentheses represent number of patients or number of lesions as appropriate

**Table 2:** Causes of death through 5 years

Cause	Zilver PTX RCT^a^			Zilver PTX Japan PMS
	DES	PTA/BMS	*p* value	DES
Cardiovascular	4.8% (16/336)	5.6% (8/143)	0.66	6.1% (55/904)
Cancer	4.8% (16/336)	1.4% (2/143)	0.11	3.0% (27/904)
Pulmonary	1.8% (6/336)	1.4% (2/143)	> 0.99	2.7% (24/904)
Stroke	0.6% (2/336)	0.7% (1/143)	> 0.99	1.5% (14/904)
Trauma/accident	0.0% (0/336)	1.4% (2/143)	0.09	0.2% (2/904)
GI	0.3% (1/336)	0.0% (0/143)	> 0.99	0.2% (2/904)
Infection	0.0% (0/336)	0.0% (0/143)	> 0.99	0.2% (2/904)
Renal	0.0% (0/336)	0.0% (0/143)	> 0.99	0.8% (7/904)
Multiple	0.3% (1/336)	0.7% (1/143)	0.51	1.5% (14/904)
Unknown	1.8% (6/336)	0.7% (1/143)	0.68	4.3% (39/904)

^a^Cause of death was only available for the 65 deaths reported during active study follow-up

**Fig. 3:**
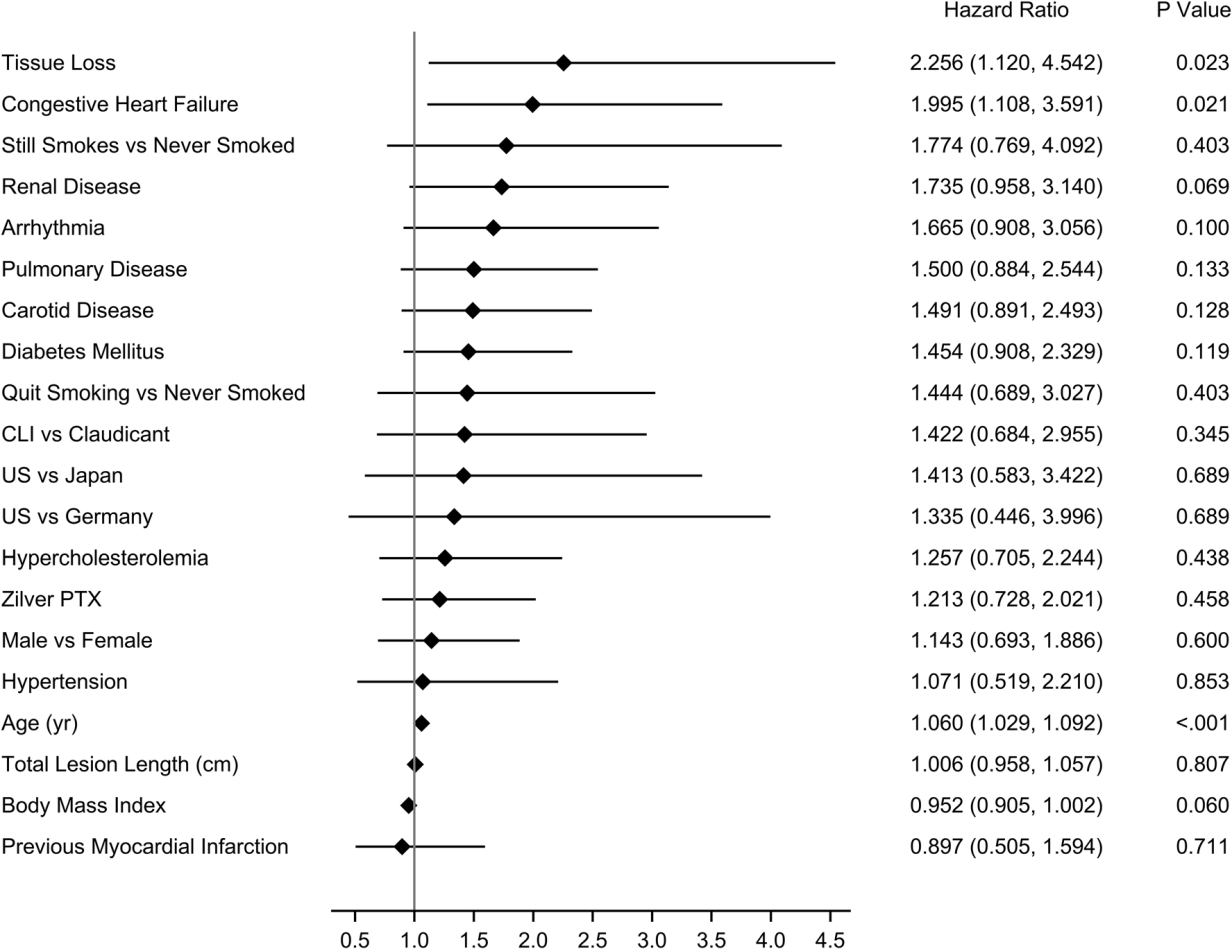
Covariate analysis for treatment in the Zilver PTX RCT. Cox proportional hazards model for mortality through 5 years. The diamonds indicate the hazard ratios, and the lines indicate the 95% confidence intervals. RCT, randomized controlled trial

The Cox proportional hazards model revealed that age (*p *< 0.001), tissue loss (*p *= 0.02), and congestive heart failure (*p *= 0.02) were significantly associated with mortality through 5 years (Fig. 3). Treatment with Zilver PTX (*p *= 0.46) was not associated with mortality. In the covariate analysis of paclitaxel dose, the significant factors were identical to the treatment analysis, and there was no association or trend of paclitaxel dose (*p *= 0.86) with mortality (Online Resource 3).

### Japan Post-market Surveillance Study

The Japan DES PMS enrolled 904 DES patients, and the Japan BMS PMS enrolled 190 BMS patients. Baseline patient and lesion characteristics for these patient groups are summarized in Table 3.

**Table 3:** Japan PMS demographics and baseline lesion characteristics

Characteristic	DES	BMS	*p* value
Age (years)	73.5 ± 8.5 (904)	73.8 ± 8.7 (190)	0.68
Gender			0.79
Male	70.2% (635/904)	71.6% (136/190)	
Female	29.8% (269/904)	28.4% (54/190)	
Diabetes	58.7% (531/904)	48.9% (93/190)	0.015
Diabetes type			0.28
Type I	8.3% (43/518)	4.4% (4/90)	
Type II	91.7% (475/518)	95.6% (86/90)	
Hypercholesterolemia	61.0% (551/904)	50.0% (95/190)	0.006
Hypertension	85.5% (773/904)	78.4% (149/190)	0.021
Carotid disease	58.3% (527/904)	45.3% (86/190)	0.001
Renal disease	43.6% (394/904)	40.5% (77/190)	0.47
Chronic renal failure	35.8% (323/903)	34.7% (66/190)	0.80
Pulmonary disease	7.5% (68/904)	6.8% (13/190)	0.88
Smoking status			0.11
Never	36.5% (330/904)	33.2% (63/190)	
Quit	45.0% (407/904)	41.6% (79/190)	
Still smokes	18.5% (167/904)	25.3% (48/190)	
Rutherford 0–3	77.9% (663/851)	65.7% (115/175)	< 0.001
Rutherford 4–6	22.1% (188/851)	34.3% (60/175)	
Lesion length (mm)	14.6 ± 9.6 (1088)	11.1 ± 8.4 (202)	< 0.001
Total occlusion	41.3% (450/1089)	35.1% (71/202)	0.10
RVD (mm)	5.7 ± 0.9 (1088)	5.7 ± 0.9 (202)	0.77
Percent diameter stenosis (%)	91.7 ± 10.8 (1089)	91.6 ± 10.1 (202)	0.93
Calcification			0.021
None	26.8% (292/1089)	29.2% (59/202)	
Little	34.9% (380/1089)	41.6% (84/202)	
Moderate	21.0% (229/1089)	19.8% (40/202)	
Severe	17.3% (188/1089)	9.4% (19/202)	
Patent runoff vessels			0.34
0	6.6% (71/1083)	10.0% (20/201)	
1	31.9% (345/1083)	31.3% (63/201)	
2	32.7% (354/1083)	29.4% (59/201)	
3	28.9% (313/1083)	29.4% (59/201)	
In-stent restenosis	18.9% (206/1089)	7.9% (16/202)	< 0.001

Numbers in parentheses represent number of patients or number of lesions as appropriate

**Fig. 4:**
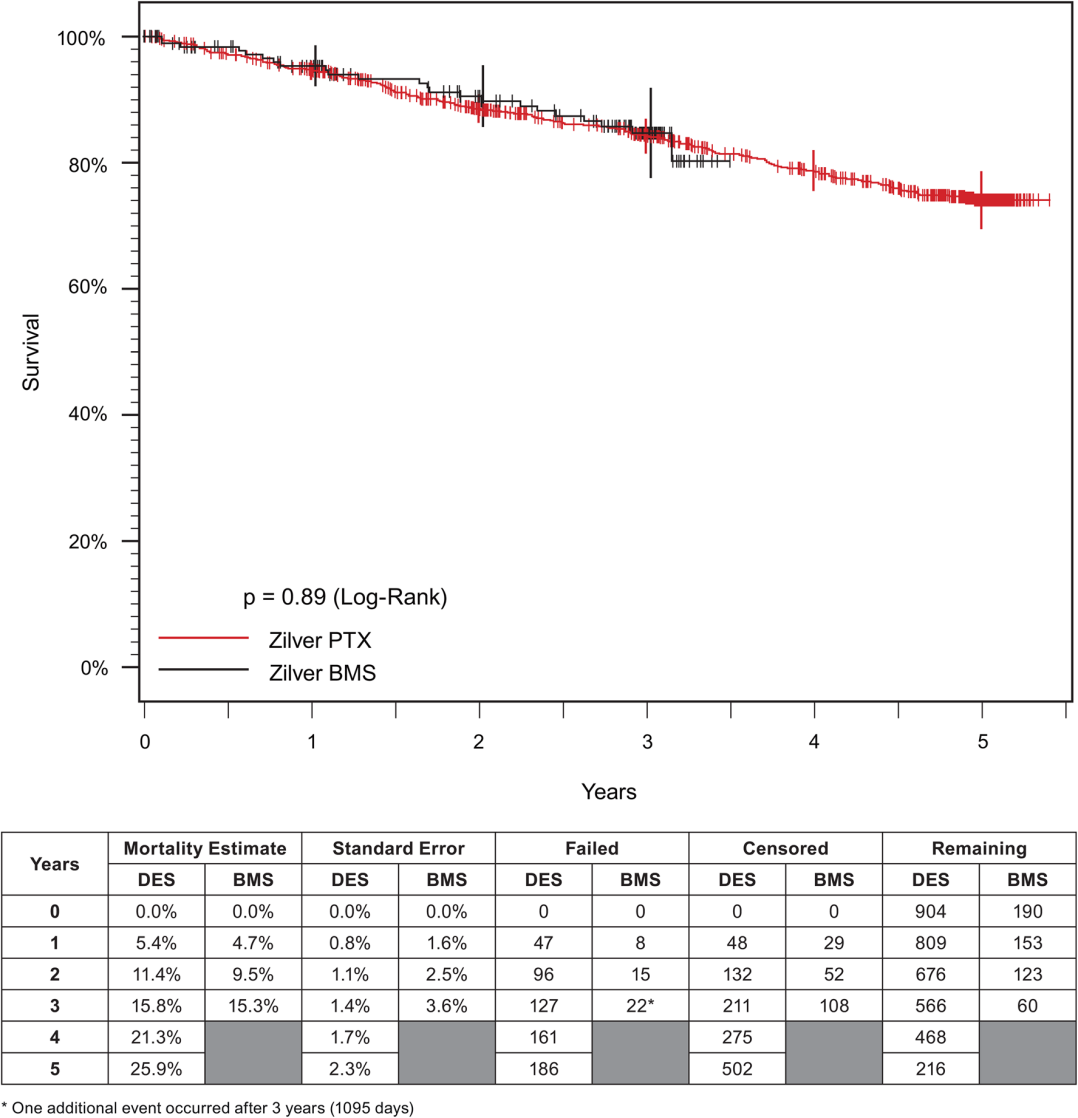
Kaplan–Meier survival analysis for patients in the Japan PMS. Long-term mortality analysis shows no difference between the DES (red curve) and BMS (black curve). PMS, post-market study; DES, drug-eluting stent; BMS, bare metal stent

In the DES group, there were 127 deaths through 3 years and 186 deaths through 5 years. In the BMS group, there were 22 deaths through 3 years. Through 3 years, the risk of mortality was 15.8% for DES group and 15.3% for BMS group. Through 5 years, the risk of mortality was 25.9% for the DES group. There was no difference in mortality between the two groups (log-rank *p *= 0.89; Fig. 4).

**Fig. 5:**
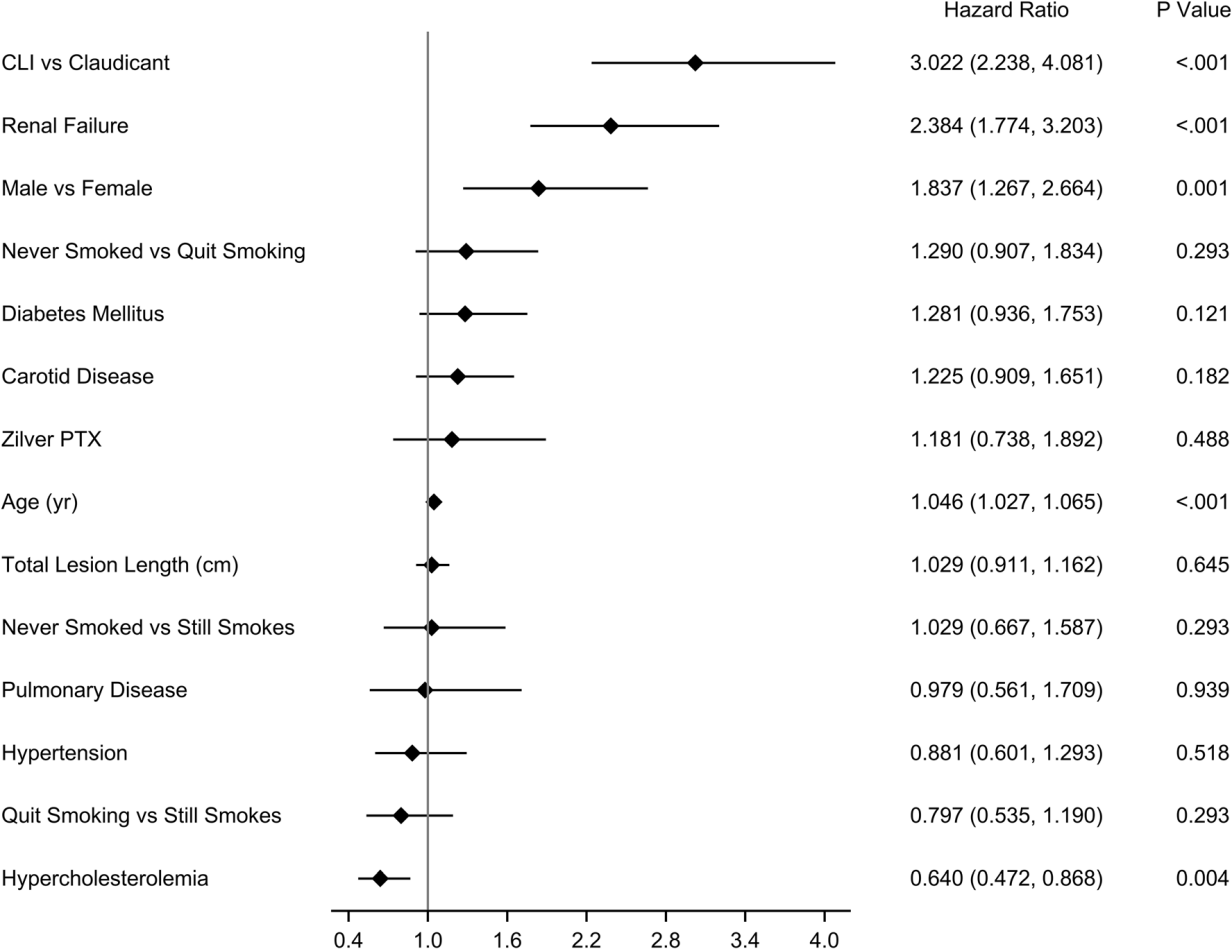
Covariate analysis for treatment in Japan PMS. Cox proportional hazards model for mortality through 5 years. The diamonds indicate the hazard ratios, and the lines indicate the 95% confidence intervals. PMS, post-market study

The Cox proportional hazards model revealed that critical limb ischemia (CLI, *p *< 0.001), age (*p *< 0.001), renal failure (*p *< 0.001), and gender (*p *= 0.001) were significantly associated with mortality (Fig. 5). Hypercholesterolemia (*p *= 0.004) was associated with lower risk of mortality. Treatment with Zilver PTX (*p *= 0.49) was not associated with mortality. In the covariate analysis of paclitaxel dose, the significant factors were identical to the treatment analysis, and there was no association or trend of paclitaxel dose (*p *= 0.07) with mortality (Online Resource 4). Table 2 shows the causes of death in the DES group through 5 years.

## Discussion

Recently, Katsanos et al. [12] published a meta-analysis of 28 RCTs (24 DCB and 4 DES) that raised concerns regarding the long-term safety of paclitaxel-coated devices. The findings have undoubtedly had a major impact on the clinical management of patients with PAD, specifically regarding the choice of therapies for lower extremity revascularization [15, 16]. The included RCTs varied in patient populations, with potentially unequal comorbidities, as well as differences in control group therapies. Although the meta-analysis pooled results for DES and DCB, there are important device-specific differences. The Zilver PTX DES has a similar paclitaxel dose compared to other peripheral paclitaxel DES and low dose compared to DCB. The total amount of paclitaxel on each device is dependent on device dimensions and the surface area coated; specific doses are provided in manufacturer instructions for use. Additionally, the coating consists of paclitaxel only and is free of any polymer or excipient [17]. Therefore, after drug elution is complete, only a BMS remains, with no long-term paclitaxel exposure.

The meta-analysis was also limited to published reports of trial outcomes, which do not provide individual patient-level information regarding cause-specific mortality data, total paclitaxel dose delivered, follow-up, or exposure to paclitaxel from contralateral endovascular treatment of PAD or protocol-defined re-intervention. These limitations bring into question the poolability of the RCTs and have the potential to alter the findings of the meta-analysis.

The previously published Zilver PTX RCT data reported 5-year all-cause mortality for only the primary randomization intent-to-treat groups [5]. However, 40% of the patients included in the PTA group in that mortality analysis were actually treated with the DES as part of protocol-defined secondary randomization and crossover. Therefore, conclusions regarding a potential association of paclitaxel with long-term mortality based on these results are flawed. This highlights the importance of relying on patient-level data rather than summary data, a limitation of the meta-analysis that has been noted by others [18–20]. Additionally, missing data for patients who did not complete the study were a limitation of previous analyses. Vital status data are now available for 94% of the patients to address this limitation.

The RCT protocol allowed placement of the DES for treatment of procedural PTA failure or for re-intervention of non-paclitaxel-treated lesions within the first year. As a result, 63 PTA patients were treated with the DES at the time of the procedure. An additional 31 patients failed their initial non-paclitaxel therapy and crossed over to treatment with DES at a median of 183 days. As defined in the study protocol, these crossover patients were then followed for the remaining 4–5 years of the study as DES patients. The published meta-analysis includes patients secondarily treated with paclitaxel devices in the PTA control group; this fundamental flaw confounds the interpretation of the results.

When evaluating the association of a drug or device with increased mortality, the most appropriate group of patients to analyze should be considered. This approach is supported by internationally harmonized guidelines endorsed by numerous regulatory bodies that state, “For the overall safety and tolerability assessment, the set of subjects to be summarized is usually defined as those subjects who received at least one dose of the investigational drug” [21]. Analyses presented in RCTs favor a conservative intent-to-treat approach to evaluate effectiveness. However, this approach cannot be used to analyze safety in this case because of a protocol-defined secondary randomization and crossover to treatment with DES, which together accounts for 40% of the patients originally randomized to PTA. The appropriate analysis compares patients who actually received the DES to patients who were not treated with the DES during the study; the analyses in this paper are consistent with these recommendations to evaluate actual treatment. The criticism that this approach results in a non-randomized comparison can be accounted for by using statistical methods such as a Cox proportional hazards model.

To address the limitation of the non-randomized comparison of DES and PTA/BMS patients, we sought to identify which factors were associated with mortality while also accounting for potential differences between patient characteristics. As expected, age and other comorbidities common in PAD patients were significantly associated with an increased risk of mortality, whereas treatment with the DES was not associated with an increased mortality risk in either the RCT or Japan PMS.

Dose escalation studies have shown that increasing dose corresponds to an increased response [22]. The authors of the recent meta-analysis proposed a relationship between paclitaxel dose and excess mortality [12]. A retrospective study revealed that mortality risk following application of the DES did not increase over 5 years, irrespective of the paclitaxel dose [23]. Despite the very low dose and transient systemic and tissue levels of paclitaxel following DES implantation, a Cox model that included total paclitaxel dose received by the patient was performed. In this analysis, the significant factors associated with an increased risk of mortality were identical to the factors identified in the treatment analysis. In both the RCT and Japan PMS, paclitaxel dose was not significantly associated with mortality. Therefore, a relationship between paclitaxel and late mortality cannot be deemed causative. Due to the post-market design of the Japan PMS, more limited data were collected on patient demographics and comorbidities than may be collected in a pre-market study. As a result, important factors relating to long-term patient mortality across a broad patient population may not have been fully identified.

In comparison with another study that enrolled Japanese PAD patients [24], the mortality rate through 5 years in the Japan DES PMS was comparable despite enrolling patients with more severe comorbidities, suggesting that mortality is a result of the natural progression of PAD and not any paclitaxel-specific effect. Although the mortality rate in the Japan DES and BMS PMS was higher than in the RCT, the RCT included a limited patient population. In clinical practice, patients with a wide range of disease are treated; therefore, the safety information from the large Japan PMS, that showed no difference for paclitaxel and non-paclitaxel-eluting devices, is more representative of patients treated in standard clinical practice.

The TransAtlantic Inter-Society Consensus (TASC) document reports a 5-year all-cause mortality rate of approximately 30% for patients with intermittent claudication [25]. Similarly, a retrospective review of intermittent claudicants who underwent endovascular revascularization reported a 5-year all-cause mortality rate of 24% [26]. Heikkila et al. [27] reported a 5-year all-cause mortality rate of 24.7% for 26,579 patients with intermittent claudication who underwent endovascular revascularization. In a meta-analysis by Sigvant et al. [28], the authors reported a 5-year all-cause mortality rate of 27% for 57,322 patients with symptomatic PAD (90% with intermittent claudication, 10% with CLI). Secemsky et al. [29] recently published a report that evaluated 51,456 patients in a US Centers for Medicare and Medicaid Services database who underwent peripheral artery stenting; 59.7% had CLI. Through 4.1 years, the mortality rate for patients treated with DES was not significantly different compared to patients treated with BMS (51.7% vs. 50.1%). The mortality rate in the Japan PMS, which included 22% of patients with CLI, was similar to the published rates and may be a reliable indicator of long-term mortality rates in the PAD patient population. The lower mortality rate in the RCT may reflect the more controlled clinical study environment.

In the Zilver PTX RCT, the most prevalent causes of death were cardiovascular disease and cancer. Although the rate of cardiovascular disease-related deaths was higher in the PTA/BMS group and the rate of cancer-related deaths was higher in the DES group, neither of these differences was significant. McDermott et al. [30] reported a 3-year cancer mortality rate of 3.0% in a cohort of 1314 PAD patients, corresponding to a 1-year annualized rate of 1.0% (i.e., approximately 5% through 5 years). Rantner et al. [31] reported that cancer was the leading cause of death in a population of 255 intermittent claudication patients, occurring in 3.9% of patients (10/255) within 5 years. These published rates for cancer-related mortality in PAD patients are comparable to the rates seen in the Zilver PTX RCT (4.8%) and Japan PMS (3.0%) studies through 5 years.

As discussed, the patient populations from the RCT and Japan PMS studies are different. Therefore, these data could not be pooled for analysis. When evaluated independently, neither study shows a significant increase in mortality for patients treated with the DES versus those not treated with the DES.

Unlike the Japan DES PMS, the Japan BMS PMS was limited to 3-year follow-up. However, there was no difference in mortality between DES and BMS patients. Both groups have an approximately 5% annual mortality rate over the available follow-up periods with no apparent increase in the annual mortality rate beyond 3 years with the DES.

During enrollment and follow-up of the RCT and Japan PMS, other peripheral paclitaxel devices were not available in the USA or Japan. Therefore, the potential confounding effect of paclitaxel use in the control group for re-intervention or treatment of the contralateral leg beyond the study protocol is minimal for these studies.

In summary, analyses of the paclitaxel-coated Zilver PTX DES utilizing patient-level data from the Zilver PTX RCT and Japan PMS demonstrated no increase in long-term all-cause mortality. Neither of these datasets substantiated the signal described by Katsanos et al. [12].

## Electronic supplementary material

Below is the link to the electronic supplementary material.

Supplementary material 1 (DOCX 15 kb)

Online Resource 3 Covariate analysis for paclitaxel dose in the Zilver PTX RCT. Cox proportional hazards model for mortality through 5 years. The diamonds indicate the hazard ratios, and the lines indicate the 95% confidence intervals. RCT, randomized controlled trial (TIFF 140,673 kb)

Online Resource 4 Covariate analysis for paclitaxel dose in the Japan PMS. Cox proportional hazards model for mortality through 5 years. The diamonds indicate the hazard ratios, and the lines indicate the 95% confidence intervals. PMS, post-market study (TIFF 140,673 kb)
