# Supplementary material for: Paclitaxel-Coated Zilver PTX Drug-Eluting Stent Treatment Does Not Result in Increased Long-Term All-Cause Mortality Compared to Uncoated Devices
Source: Cardiovasc Intervent Radiol. 2019 Sep 9;43(1):8–19. doi: 10.1007/s00270-019-02324-4 (PMC6940321; doi:10.1007/s00270-019-02324-4)
Supplement: Supplementary file 1 — Supplementary material 1 (DOCX 15 kb) [file 270_2019_2324_MOESM1_ESM.docx]

**Online Resource 1. Study characteristics for the Zilver PTX RCT, Japan DES PMS, and Japan BMS PMS**

|  | **Study** | | |
| --- | --- | --- | --- |
|  | **Zilver PTX RCT** | **Japan DES PMS** | **Japan BMS PMS** |
| Device(s) | - Zilver PTX DES - PTA - BMS | - Zilver PTX DES | - Zilver BMS |
| Region(s) | US, Germany, Japan | Japan | Japan |
| Design | - Investigational - Primary randomization to DES or PTA - Secondary randomization to DES or BMS - Cross over to DES within first year | - Post-market - Consecutive enrollment of all patients treated with Zilver PTX DES | - Post-market - Consecutive enrollment of all patients treated with Zilver BMS |
| Number of Patients | 479 | 904 | 208^a^ |
| Key Inclusion Criteria | - Rutherford category ≥2 - ≥50% diameter stenosis - Reference vessel diameter 4 to 9 mm - Lesion length ≤14 cm - At least 1 patent runoff vessel | - Treatment with Zilver PTX DES | - Treatment with Zilver BMS |
| Key Exclusion Criteria | - >50% stenosis of the inflow tract - Previous target vessel stenting | - None | - None |
| Follow-up | 5 years | 5 years | 3 years |
| Status | Study complete | Study complete | Study complete |

^a^ Patients who were enrolled in the BMS study but who also had a DES placed (n=18) were excluded from the current analysis

**Online Resource 2. Adverse events in the Zilver PTX RCT through 5 years**

| **Event Category** | **DES** | **PTA / BMS** | ***p*-value** |
| --- | --- | --- | --- |
| Cardiovascular | 36.9% (124/336) | 37.8% (54/143) | 0.92 |
| Pulmonary | 19.3% (65/336) | 21.0% (30/143) | 0.71 |
| Renal | 14.3% (48/336) | 13.3% (19/143) | 0.89 |
| Gastrointestinal | 19.3% (65/336) | 16.8% (24/143) | 0.61 |
| Wound | 9.2% (31/336) | 9.1% (13/143) | >0.99 |
| Vascular | 71.4% (240/336) | 70.6% (101/143) | 0.91 |
| Miscellaneous | 55.4% (186/336) | 55.9% (80/143) | 0.92 |
